# Case of Parturition

**Published:** 1856-12

**Authors:** J. Beerbower

**Affiliations:** Mt. Ayr, Iowa


### CASE OF PARTURITION.—SPONTANEOUS PELVIC EVOLUTION.

BY J. BEERBOWER, M. D., OF MT. AYR, IOWA.

Owing principally to the peculiar circumstances under which I was placed, the following case was at the time one of deep interest to me, and thinking it might be so to some extent to the profession at large, I offer it for the pages of the *Iowa Medical Journal.*

On the morning of the 13th of June last, I was summoned, in great haste, to see a Mrs. S., (represented as dying) in labor, with her third child. I arrived at the house about 6 oclock, A. M. On entering the lying-in room, my first impression was that the messenger's apprehensions were not without reason—that the woman was actually dead. The grief of the husband and female attendants present, seemed to confirm this impression. The attending accoucheur having retired to bed, I learned, on enquiry, that the woman had been in actual labor for 72 hours, though her pains had altogether suspended during the entire preceding night, and she had frequently vomited. I approached the bed, and found her in the following deplorable condition: pulse almost imperceptible; tongue coated; some sordes on the teeth; feet and hands quite cold; respiration hurried.

On examination, per vaginam, I found the child in the left cephaloiliac position — the right arm protruding the entire length, head placed in the left iliac fossa, breech in the right, its dorsal plane in front. On the part of the mother, there were great retention of urine, and bowels loaded; no attempt having been made to relieve either during the 72 hours.

Supposing the exhaustion in this case to be more apparent than real; caused partly by the surrounding depressing circumstances and partly by the long continued efforts of the uterus, loaded bowels, bladder, &c., (as the effects of the treatment adopted, proved beyond a doubt, to have been the case,) my first efforts were to inspire her with hope. In the accomplishment of this, I had but little difficulty; my presence alone, though an entire stranger, seemed to animate and give her encouragement. Very soon I had the pleasure of seeing a reaction—the pulse rose full and strong, the extremities became warm, &c. Nature was again aroused from her lethargy to execute and fulfil her work. Her efforts, although the most vigorous I ever witnessed, seemed to be unavailing. Instead of presenting the aspect of a corpse, the patient now looked like a robust plethoric woman,—florid countenance; muscles tense and rigid, being naturally of a good constitution, medium size and squarely built. The pelvis being of the ordinary size and well proportioned, I now determined to attempt version, although fruitless attempts had been made by the attending accoucheur. Accordingly I opened a vein and allowed the blood to flow freely from a large orifice, until four pints were taken; she now manifested some nausea, I then administered an active cathartic, followed in due time by enemata. This was succeeded by frequent nauseating doses of tartar emetic, and ipecac. After procuring the necessary evacuations, I prescribed sulph. morphiæ, in gr. ss doses, every twenty minutes, at the same time allowing her the free use of chloroform, which she clung to with avidity, begging and imploring for it when withheld. Notwithstanding all my antagonistical efforts, which only seemed to encourage and stimulate the woman, the uterine contractions continued with apparently increased energy. A movement of rotation occurred by which the child's long axis was brought very nearly into an antero-posterior direction, its cephalic extremity placed above the horizontal branch of the pubis. The trunk being now bent double, was forced *en masse* into the excavation; the head now being arrested in consequence of the shortness of the neck, the uterine efforts acting on the pelvic extremity, forced it through into the external world: after which the patient was, before 10 o'clock, A. M., easily delivered of a fully developed dead male child, which measured, in length, 27 inches; its weight I did not ascertain.

The placenta in this case, was preternaturally large, independently of the congestion, and was rather firmly adherent to the fundus and left parietes of the uterus, although detached without any particular difficulty. The uterus contracted naturally, and the woman convalesced rapidly.

